# Influence of arm position during infraclavicular subclavian vein catheterization in coronary artery bypass graft surgery

**DOI:** 10.15171/jcvtr.2018.33

**Published:** 2018-12-05

**Authors:** Masoud Tarbiat, Maryam Davoudi, Sayed Ahmadreza Salimbahrami

**Affiliations:** Clinical Research Development Unit of Farshchian Heart Center, Department of Anesthesiology, School of Medicine, Hamadan University of Medical Sciences, Hamadan, Iran

**Keywords:** Catheterization, Complications, Coronary Artery Bypass, Subclavian Vein

## Abstract

***Introduction:*** Percutaneous subclavian vein catheterization via infraclavicular approach is one of the most widely used cannulation techniques for inserting catheters into a central vein. The aim of this study was to evaluate influence of arm position during infraclavicular subclavian vein catheterization with landmark-based technique in coronary artery bypass graft (CABG) surgery.

***Methods:*** Between September 2017 and June 2018, this prospective randomized clinical trial was performed in 320 patients. The patients were randomly assigned to the Neutral group (the arms kept by the side) or Abduction group (the arm was abducted to 90°). The success and complication rates were compared in the two groups. The data were analyzed using SPSS software.

***Results:*** In the first attempt of subclavian vein cannulation, the success rate had no significant difference between the two groups (*P* = 0.185). In the second attempt of catheterization, the success rate in Abduction group (40.5%) was lower than Neutral group (81.2%). The overall success rate in two attempts were (84.4%) in the Abduction group and (96.2%) in the Neutral group. There was a significant difference between two groups in the second and overall success rates (*P * = 0.0001). In 34 (10.6%) patients, subclavian artery puncture occurred, 30 (18.8%) in the Abduction group and 4 (2.5%) in the Neutral group. There was a significant difference between two groups (*P * = 0.0001). Pneumothorax was occurred in 15 (9.4%) in the Abduction group and 3 (1.9%) in the Neutral group. There was also a significant difference between two groups (*P * = 0.004). The differences in other complications on two groups were statistically insignificant.

***Conclusion:*** Compared with Abduction group, the Neutral group resulted in higher success rate and fewer subclavian artery puncture and pneumothorax. The incidences of other complications were similar on both groups.

## Introduction

Central vein catheterization is a standard of care during cardiac surgery for monitoring central venous pressure, drug administration, cardiopulmonary resuscitation, rapid fluid resuscitation, difficult peripheral catheterization and insertion of a transvenous pacemaker.^1,2^ Percutaneous subclavian vein catheterization is a well-known method of central venous catheterization and infraclavicular approach is the most common technique used for it. The common arm position during infraclavicular approach is neutral position (the patient’s ipsilateral arm is placed at the side). Recently, a few studies reported that placing the arm in 90° abduction from the trunk improve ultrasound visualization of the subclavian vein and minimize the risk of misplacement of the catheter during infraclavicular approach.^3,4^ Therefore, this study investigated the influence of arm position during infraclavicular subclavian vein catheterization with landmark-based technique.

Although some of the physicians believe internal jugular vein catheterization is better than subclavian vein catheterization owing to a lower risk of pneumothorax and a high success rate of cannulation, accidental carotid artery puncture in these high-risk atherosclerotic patients is a serious complication. Moreover, subclavian venous catheterization has advantages of lower risk of infection and improving patient comfort especially for cardiac patients who often have a catheter in their neck for at least a week.^5,6^

Therefore, in our institute for improving patient comfort, nursing care and ease of neck motion, subclavian vein catheterization is preferred.

## Materials and Methods

This study was a prospective randomized clinical trial that was carried out between September 2017 and June 2018 after approval from the ethics committee. Written informed consent was obtained from all the patients before the study. Three hundred and Twenty patients of the American Society of Anesthesiologists physical status II-III, aged between 25 and 89 years scheduled to undergo coronary artery bypass graft surgery (CABG) were enrolled for the study. Our exclusion criteria were emergency surgery, prior infection or radiotherapy over the puncture site, prior pneumothorax, blood coagulopathy, previous thoracotomy or right subclavian vein catheterization and history of clavicular fracture. Routine assessment before subclavian vein catheterization were platelet count, prothrombin time (PT), partial thromboplastin time (PTT) and chest X-ray.

**Figure 1:**
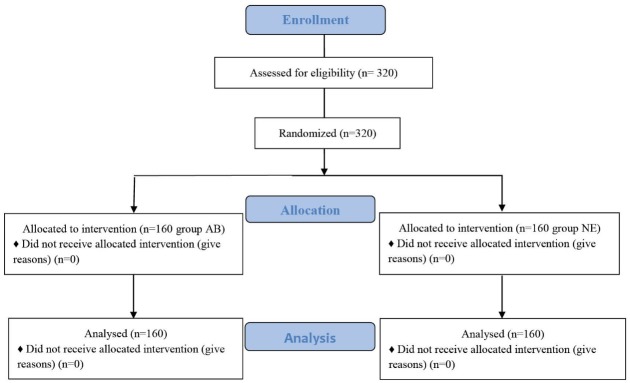
Flow chart of the patients enrolled in the study.

The patients were randomly assigned to one of the two groups [arm abducted to 90° (AB) or arms kept by the side (NE)] of the trial using block randomization method (size of block = 4, list made up of 80 blocks of four allocations, with two treatment and two control allocations randomly ordered within each block). The flow diagram presented in Figure 1.

Allocation was concealed with numbered, sealed and opaque envelopes containing the group allocation cards by an independent enrolling investigator.

The blindness of practitioner was not possible; however, patients and statistical analyzer were blind because they could not distinguish between two types of procedure.

After induction of anesthesia, tracheal intubation and, the head of the patient was rotated slightly to the contralateral side and the arm was abducted to 90° (AB) or the arms kept by the side (NE). All patients were disconnected from mechanical ventilation during needle insertion. The cannulation of right subclavian veins were done in the Trendelenburg position by right-handed physicians. The puncture site of subclavian vein was below the midpoint of the clavicle. After puncture of the subclavian vein, the modified Seldinger technique was used for all catheterization procedures. The depth of triple lumen central venous catheter insertion was 15 cm for all catheterization. After successful insertion, all 3 lumens were checked for blood aspiration, and if aspiration was unsuccessful, the catheter was pulled back slowly to the point that allows free blood aspiration and fixed at that level. If the assigned approach was unsuccessful after two attempts (each skin puncture was defined as an attempt) at subclavian vein cannulation, the right internal jugular vein was chosen for catheterization. Immediately after the surgery, chest radiographs were obtained to evaluate the complications (pneumothorax, hemothorax) and position of the catheter tip in the intensive care unit.

Data collected included age, sex, height, weight, body mass index, arm position on catheter placement, number of attempts to cannulate vein, success rate of catheterization, arterial puncture, pneumothorax, haemothorax, hematoma at puncture site, thoracic duct damage and malposition of the catheter tip. It should be noted that we did not place anything longitudinally beneath the vertebral column between the scapulae during procedure.

The sample size based on similar study with a power of 0.9 and statistical significance of 0.05 was 159 patients in each group. However, we decided to enroll 160 patients in each group.

Kolmogorov–Smirnov normality test revealed the normality continues data. The Chi-square test was used to assess the relationship between success rates of cannulation and arm position on catheter placement in two groups. Moreover, *t* test was carried out for assessment of the differences between means of continues variables. The *P***≤0.05** indicated statistical significance. All statistical calculations were performed using SPSS version 16 software.

## Results

A total of 320 CABG surgery patients were enrolled in present study. The patients’ demographic characteristics are shown in Table 1. They were randomly separated into two equal groups. The right subclavian veins of each group of patients (n = 160) were cannulated with the arm on AB or NE position .

**Table 1:** Patient characteristics

**Variable**	**Abduction Group (n = 160)**	**Neutral Group (n = 160)**	**Total n=320**	***P*** ** value**
Gender ratio, male/female	110/50	120/40	230/90	0.214
Age (y) (Mean± SD)	61.38±9.73	59.91±10.75	60.65±10.25	0.202
Weight (kg) (Mean± SD)	70.96±12.06	72.62±15.05	71.79±13.64	0.218
Height (cm) (Mean± SD)	164.70±8.81	166.70±8.68	165.72±8.79	0.279
BMI (kg/m^2^)(Mean± SD)	26.16±4.14	26.02±4.21	26.09±4.17	0.481

In 246 (76.9%) patients, the first attempt at subclavian vein cannulation was successful, 118 (73.8%) in the arm on AB position and 128 (80%) in the arm on NE position. In the first attempt of subclavian vein cannulation, the success rate had no significant difference between two groups (*P *= 0.185). In 43 (58.1%) patients, the second attempt at subclavian vein cannulation was successful, 17 (40.5%) in the AB group and 26 (81.2%) in the NE group.

The overall success rate in two attempts were 135 (84.4%) in AB group and 154 (96.2%) in NE groups. There was a significant difference between two groups in the second and overall success rates (*P *= 0.0001). In 31 (9.7%) patients, subclavian vein cannulation was failed after two attempts in both groups (Table 2). In 13 (4.01%) of 320 (6 in the arm on AB position and 7 in the arm on NE position groups) catheters which placed through right subclavian vein were misplaced. In the NE group, five catheter tips were placed in the right internal jugular vein and in two, the catheter tip was placed in the contralateral subclavian vein .

**Table 2:** Success of catheterization

**Attempts**	**Variable**	**Abduction Group, No. (%)**	**Neural Group, No. (%)**	**Total, No. (%)**	**P value**
First attempt	Success	118 (73.8)	128 (80.0)	246 (76.9)	0.185
	Fail	42 (26.2)	26 (81.2)	74 (23.1)	
Second attempt‏ (n=74)	Success	17 (40.5)	26 (81.2)	43 (58.1)	0.0001
	Fail	25 (59.5)	6 (18.8)	31 (41.9)	
Total	Success	135 (84.4)	154 (96.2)	289 (90.3)	0.0001
	Fail	25 (15.6)	6 (3.8)	31 (9.7)	

**Figure 2:**
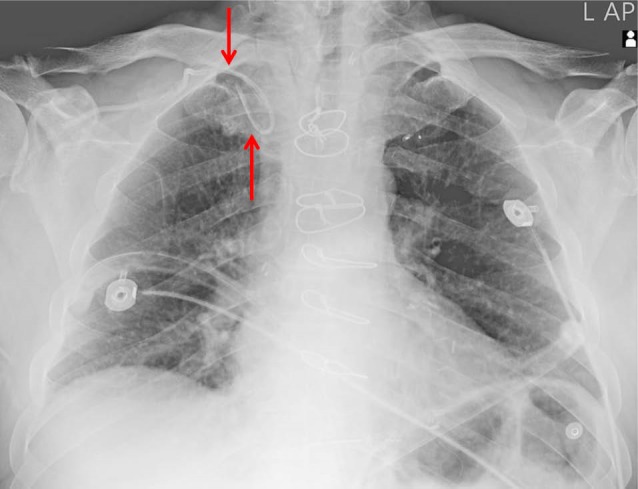
Postoperative chest X-ray depicting right subclavian vein catheter formed a loop around itself over the right subclavian vein in abduction arm position (arrows).

In the AB group, four catheter tips were placed in the right internal jugular vein, one catheter formed a loop around itself over the right subclavian vein (Figure 2) and another one catheter tip was placed in the contralateral subclavian vein. There was no significant difference in malposition of the catheter tips between two groups (*P *= 0.77).

In 34 (10.6%) patients, subclavian arterial puncture occurred, 30(18.8%) in the AB group and 4 (2.5%) in the NE group. The overall incidence of subclavian artery puncture was 10.6%. There was a significant difference between two groups (*P*=0.0001).

Pneumothorax was occurred in 15 (9.4%) in AB group and 3(1.9%) in the NE group. The overall incidence of pneumothorax was 5.6%. There was also a significant difference between two groups (*P*= 0.004).

There was no significant difference between two groups in other complications (Table 3).

**Table 3:** Complications of catheterization

**Complications**	**Abduction Group** **No. (%)**	**Neutral Group** **No. (%)**	**Total** **No. (%)**	**P value**
Malposition	6 (3.80)	7 (4.40)	13 (4.01)	0.77
Subclavian artery puncture	30 (8.80)	4 (2.50)	34 (10.60)	0.0001
Pneumothorax	15 (9.40)	3 (1.90)	18 (5.60)	0.004
Hematoma at puncture site	1 (0.60)	0 (0.00)	1 (0.30)	0.31

## Discussion

One of the most widely used cannulation techniques for inserting catheters into a central vein is percutaneous subclavian vein catheterization via infrasubclavicular approach.

This procedure is associated with some complications which may be life threatening (cardiac tamponade, pneumothorax, hemothorax, subclavian arterial puncture).^7,8^

Therefore, it is very important to optimize the procedure of subclavian vein cannulation and maximize the chance *of* successful catheter placement.^9,10^

In 2016, Sadek and colleagues suggested that placing the arm in 90° abduction could improve ultrasound visualization of the subclavian vein and could be an alternative approach compared with standard techniques.^3^

In 2016, Ahn and colleagues^4^ also documented that upper arm abduction may minimize the risk of misplacement of the catheter during real-time ultrasound-guide infraclavicular venous catheterization. They stated that the success rate of catheterization 97.1%in the NE group and 98.8% in the AB group. There was no significant difference between two groups. Previous studies have documented overall success rate of the infraclavicular subclavian vein *catheterization* ranging from 84% to 97%.^5^ Kim et al reported a 95.6% success rates for infraclavicular subclavian vein cannulation in elective surgery patients with the landmark-based technique.^11^ In cardiac surgery, the success rates of subclavian vein catheterization with using landmark-based technique have been reported 97% to98%.^2,5,6^ In Oh and his colleagues study, the incidence of successful placement of catheter with using ultrasound and landmark-based methods in neurosurgery patients were 87% and 56%, respectively.^12^ This obvious difference was perhaps due to possibility of the advantage of lateral approach in ultrasound group as a consequence of the insertion site, *small sample size of study* participants and *difference in physician skill* level. In this study, the overall success rates in two attempts were 84.4%in the AB group and 96.2% in the NE group. There was a significant difference between two groups in the overall success rates (*P*=0.0001).

These results are not in concordance with the study of Ahn et al which reported that there are no significant differences between the success rate in the AB and NE groups (98.8% versus 97.1%, respectively). This obvious difference was perhaps due to different techniques (real-time ultrasound-guide versus landmark-based) and insertion site of cannulation.

Catheter tip malpositioning is one of the most frequent complications of subclavian vein catheterization. A malpositioned catheter tip causes inaccurate central venous pressure reading. Thrombosis of internal jugular vein, elevated intracranial pressure, retrograde perfusion of the intracranial veins and infection are other complications which may occur.^13,14^

During subclavian vein catheterization, the most common misplacement of the catheter is cephalad, into the ipsilateral internal jugular vein (about 60% of all malpositioning).^15^

The cause of higher rate of malpositioning catheter tip into jugular vein in right-sided subclavian vein catheterization is sharp angulation of it with the right internal jugular vein, so catheter tip may collides with the medial wall of the right brachiocephalic vein.^9^ In cardiac surgery, catheter tip malpositioning of subclavian vein catheterization with using landmark-based technique have been reported 3.2% to 9.6%.^2,5,6^ In Oh and colleagues study, the incidence of misplacement of catheter tip during subclavian vein catheterization were 11% and 7% with using real-time ultrasound-guide and landmark-based technique , respectively. Ahn and colleagues documented a 3.9% malpositioning catheter in NE group and 0.4% malpositioning catheter in AB group with using real-time ultrasound-guided subclavian vein cannulation. There was a significant difference between two groups (*P*=0. 01)

In present study, there was no significant difference between two groups for malposition of the catheter tip (3.8 % in NE group versus 4.4% in AB group).

Inadvertent puncture of the subclavian artery is another well-known complication of the subclavian vein cannulation. The right subclavian-jugular venous junction overlies the subclavian artery, causing this artery more susceptible to damage than the left subclavian artery.^16^ In Ahn and colleagues study, there was no significant difference between two groups for subclavian artery puncture with using real-time ultrasound-guide. Unlike previous study, the incidence of inadvertent subclavian artery puncture in AB group was significantly higher than in the NE group with using landmark-based technique in present study (18.8% versus 4%, respectively).

Although Ahn and et al reported no Pneumothorax in their study, the incidence of pneumothorax in AB group was higher than in the NE group in our study (9.4% versus 1.9%, respectively).

It is notable that although real-time ultrasound-guided and pocus (point-of-care ultrasound) possess higher success rate and efficiency, they are not available in all clinical institutes like our medical facility. Real-time ultrasound-guided is technically difficult for some of physicians and requires training.^1^ It is a *time*-*consuming procedure.* Anyway, subclavian vein catheterization with using landmark-based technique is still a choice method for some of clinicians. Moreover, all physicians probably must know several techniques including the landmark-based technique for subclavian vein catheterization.

Finally, in our opinion, 18.8% arterial puncture rate and 9.4% pneumothorax rate (in AB group) are too high. Therefore, subclavian vein catheterization with landmark-based technique at AB position is not appropriate for subclavian vein catheterization.

## Conclusion

The result of this study suggests that placing the arm in 90° abduction for infraclavicular subclavian vein catheterization would not be an appropriate alternative compared to placing the arm in side with landmark-based technique.

## Competing interests

The authors declare that they have no competing interest.

## Ethical approval

The study was approved by Ethics Committee of Hamadan University of Medical Sciences and registered under the code number IRCT2017062110348N4. This study also was registered in Iranian Registry of Clinical Trials (identifier: IRCT2017062110348N4; https://www.irct.ir/trial/10871).

## Acknowledgement

The authors gratefully acknowledge the Research Vice Chancellor of Hamadan University of Medical Sciences for all the material and support in this study.
